# Editorial to the *QHW* thematic cluster “Health, Physical Activity and Lifestyle”

**DOI:** 10.3402/qhw.v10.29156

**Published:** 2015-08-14

**Authors:** Urban Johnson, Natalia Stambulova

**Affiliations:** 1 Sport and Exercise Psychology, School of Health and Welfare, Halmstad University, Halmstad, Sweden, E-mail: urban.johnson@hh.s; 2 School of Health and Welfare, Halmstad University, Halmstad, Sweden

As guest editors of the *International Journal of Qualitative Studies on Health and Well-being's* thematic cluster on Health, Physical Activity and Lifestyle, it is our privilege to introduce the theme and six original articles included in the cluster. The papers address some central aspects of the theme (e.g., unhealthy habits and how to deal with them, exercise interventions and how to keep participants motivated and committed) from psychological, sociological, pedagogical, medical, and also interdisciplinary perspectives through implementation of different qualitative research approaches (e.g., thematic analysis and narrative analysis) and designs (e.g., single case study and interviews with intervention participants). The author team is internationally representing Sweden, Denmark, and the United Kingdom. Two review papers open the cluster and are followed by four research papers.

## Introduction to the theme

Physical activity, or the lack of it, is a major public health issue in many countries. The World Health Organization (WHO) has reported that physical inactivity is one of the ten leading causes of death in developed countries resulting in about 1.9 million preventable deaths worldwide annually (WHO, 2002). Considerable knowledge has been accumulated over the past decades concerning the importance of regular and (if needed) specified physical activity in prevention and treatment of a number of diseases. Warburton, Nicol, and Bredin (2006), for instance, based on reviewing literature on health benefits of physical activity, concluded that there is overwhelming evidence of the effectiveness of regular physical activity in the primary and secondary prevention of several chronic diseases, including cardiovascular disease and obesity. It is also well-known that disease prevention and treatment are not the only benefits of exercising. Research confirms that exercising is often paired with other components of healthy lifestyle (e.g., healthy eating habits) and contributes to health-related resources and better quality of life across different populations (e.g., Baize, Johnson, & Plotnikoff, 2007; Penedo & Dahn, 2005).

International (Haskell et al., 2007) and Swedish physical activity guidelines (Socialstyrelsen, 2005) suggest that adults aged 18–65 need moderate intensity aerobic (endurance) physical activity for a minimum of 30 minutes 5 days a week, or vigorous intensity aerobic physical activity for a minimum of 20 minutes 3 days a week (Haskell et al., 2007) to promote and maintain their health. However, far from all people of this age range follow these recommendations. Therefore, there is a need and a challenge to develop and implement intervention techniques motivating people to begin and maintain their physical activity. There is also a need to help people find the individually optimal amount, intensity, and content of physical activity to enhance their health and quality of life benefits. Specific populations (e.g., people with disabilities) and their physical activity needs should also be more in researchers’ and practitioners’ focus. Currently, across behavioral sciences the most popular approach to meet these challenges is the self-determination theory (Deci & Ryan, 2000) and relevant interventions with psychological techniques used to increase the participants’ inner interest and self-efficacy for physical activity and healthy eating (e.g., Ashford, Edmunds, & French, 2010; Greaves et al., 2011). Articles in this thematic cluster contribute to better understand these challenges and needs.

## Introduction to the papers included in the thematic cluster

In the first paper, Håman, Barker-Ruchti, Patriksson, and Lindgren (2015) provide an integrative literature review on orthorexia nervosa using current conceptualization of healthism and the “philosophy of science world map” (Gunnarsson, 2014) as their guiding frameworks. Analysis of 19 empirical and theoretical papers that met the selection criteria for this review revealed a multifaceted nature of orthorexia research, but with a majority of studies focusing on orthorexia as an individual issue (i.e., empirical-atomistic approach). The authors stated a need for further research of the phenomenon, especially from an empirical-holistic perspective to demonstrate how people's orthorexia-type of thinking is internalized from sociocultural contexts and paired with other aspects of their lifestyle (e.g., physical activity).

In the second paper, Kristén, Ivarsson, Parker, and Ziegert (2015) provide an interdisciplinary meta-review on the literature dealing with health determinants/predictors including various forms of physical activity, and—more broadly—a physically active lifestyle. Then the authors shift their focus to a review of the intervention studies that incorporated different forms of physical activity and were aimed at promoting health and well-being. In the conclusive part of the paper, they elaborate on advantages of having an interdisciplinary approach in designing health-related interventions (e.g., capturing different biopsychosocial aspects of health, using theoretical frameworks on health determinants, and employing longitudinal face-to-face intervention designs).

In the third (research) paper, Staveborg Kerkelä, Jonsson, Lindwall, and Strand (2015) provide insights from participants involved in a 6-month exercise intervention about their individual experiences of the context and content of the intervention program. Particular focus of the study was on how standardized intervention based on the self-determination theory approach might be adjusted to fit the individual needs of the participants. Semi-structured interviews were used for data collection, and thematic (inductive) analysis revealed three major (e.g., the frames of the intervention) and ten lower order themes (e.g., relationship to the coach, individualized exercise) to describe factor and strategies that can be taken into account to tailor exercise interventions.

In the fourth paper, Lindqvist, Kostenius, Gard, and Rutberg (2015) explored parents’ experiences of being a part of their adolescents’ empowerment-inspired PA intervention. Content analysis of the interview data collected from 10 parents revealed three sub-themes and one major theme. One major theme is “parents are one important part of a success for PA intervention”.

In the fifth (single case study) paper, Stelter (2015) shared his “whole-life perspective” approach in health-related coaching intervention helping a woman who tried different diets over years to relate specific events and habits in her work and everyday life to her specific (unhealthy) eating habits. The qualitative data analyzed in this paper included audio-recorded selected sessions, the coach's notes, and the coachee's reflections on the process and outcomes of the intervention. Positive changes were initiated in the coachee with regard to her eating habits, physical activity, and healthy lifestyle more in general.

In the sixth (research) paper, Papathomas, Williams, and Smith (2015) used narrative approach to understand personal meanings of exercise participation in the spinal cord injured (SCI) population. In-depth conversational interviews were conducted with 30 physically active male and female SCI individuals. The data were analyzed focusing on content of the stories but particularly on plots to identify a coherent narrative structure. Results of the analysis revealed that exercise participation was seen by this specific population as “restitution,” “medicine,” and “progressive redemption.” The authors concluded that the identified narrative typology may be a useful tool for physical activity promotion initiatives among SCI population.

Finally, as guest editors we wholeheartedly thank the authors for their tireless efforts in creating interesting and high-quality contributions, and all the reviewers for their constructive comments that contributed to raise the scientific quality of the thematic cluster. Our deep thanks also go the editors of the journal and the publisher, for smooth cooperation during the preparation process. We do hope that this thematic cluster will contribute to expanding the scope of the *International Journal of Qualitative Studies on Health and Well-being* and attract more researchers in the sport/exercise sciences to contribute to the journal in future.

*Urban Johnson* Sport and Exercise Psychology School of Health and Welfare Halmstad University, Halmstad, Sweden 
E-mail: urban.johnson@hh.s *Natalia Stambulova* School of Health and Welfare Halmstad University Halmstad, Sweden
